# Targeting PirA*^vp^* and PirB*^vp^* Toxins of *Vibrio parahaemolyticus* with Oilseed Peptides: An In Silico Approach

**DOI:** 10.3390/antibiotics10101211

**Published:** 2021-10-05

**Authors:** Joe-Hui Ong, Wey-Lim Wong, Fai-Chu Wong, Tsun-Thai Chai

**Affiliations:** 1 Department of Chemical Science, Faculty of Science, Universiti Tunku Abdul Rahman, Jalan Universiti, Bandar Barat, Kampar 31900, Perak, Malaysia; candy3997@1utar.my (J.-H.O.); wongfc@utar.edu.my (F.-C.W.); 2 Department of Biological Science, Faculty of Science, Universiti Tunku Abdul Rahman, Jalan Universiti, Bandar Barat, Kampar 31900, Perak, Malaysia; wongwl@utar.edu.my; 3 Center for Agriculture and Food Research, Universiti Tunku Abdul Rahman, Jalan Universiti, Bandar Barat, Kampar 31900, Perak, Malaysia

**Keywords:** AHPND, molecular docking, oilseed, peptide, PirA*^vp^*, PirB*^vp^*, shrimp, toxin, *Vibrio parahaemolyticus*

## Abstract

Acute hepatopancreatic necrosis disease (AHPND), caused by PirA*^vp^*- and PirB*^vp^*-releasing *Vibrio parahaemolyticus* strains, has resulted in massive mortality in shrimp aquaculture. Excessive use of antibiotics for AHPND management has led to antibiotic resistance, highlighting the urgency to search for alternatives. Using an in silico approach, we aimed to discover PirA*^vp^*/PirB*^vp^*-binding peptides from oilseed meals as alternatives to antibiotics. To search for peptides that remain intact in the shrimp digestive tract, and therefore would be available for toxin binding, we focused on peptides released from tryptic hydrolysis of 37 major proteins from seeds of hemp, pumpkin, rape, sesame, and sunflower. This yielded 809 peptides. Further screening led to 24 peptides predicted as being non-toxic to shrimp, fish, and humans, with thermal stability and low water solubility. Molecular docking on the 24 peptides revealed six dual-target peptides capable of binding to key regions responsible for complex formation on both PirA*^vp^* and PirB*^vp^*. The peptides (ISYVVQGMGISGR, LTFVVHGHALMGK, QSLGVPPQLGNACNLDNLDVLQPTETIK, ISTINSQTLPILSQLR, PQFLVGASSILR, and VQVVNHMGQK) are 1139–2977 Da in mass and 10–28 residues in length. Such peptides are potential candidates for the future development of peptide-based anti-AHPND agents which potentially mitigate *V. parahaemolyticus* pathogenesis by intercepting PirA*^vp^*/PirB*^vp^* complex formation.

## 1. Introduction

The shrimp is one of the most valuable aquaculture species consumed by people from many parts of the world. However, intensive shrimp culture and improper management have led to the outbreak of several diseases, such as acute hepatopancreatic necrosis disease (AHPND), hepatopancreatic microsporidiosis, infectious hypodermal and hematopoietic necrosis, and white spot disease [1,2,3]. AHPND, commonly known as early mortality syndrome, has caused huge loss in the whiteleg shrimp (*Litopenaeus vannamei*) industry. This bacterial shrimp disease has resulted in mass shrimp mortality and low shrimp production [3,4]. Administration of antibiotics in shrimp culture has been most widely used in the past in controlling the AHPND infection caused by *Vibrio* species [4]. Excessive use of antibiotics in shrimp culture has created antibiotic resistance in the pathogenic *Vibrio*. For instance, AHPND-causing *Vibrio campbellii* from China was found to carry multiple antibiotic resistance genes [5]. In Mexico, several *Vibrio parahaemolyticus* AHPND strains were reported to have the *tetB* gene coding tetracycline resistance [6]. The spread of antibiotic resistance genes among the pathogens may lead to the failure of AHPND control if the management of AHPND mainly depends on antibiotics. Moreover, the contamination of aquatic ecosystems by antibiotics is of immense concern to environmental and human health [7]. Thus, there is an urgent need to develop other anti-AHPND agents as alternatives to antibiotics.

*V. parahaemolyticus* is a halophilic, Gram-negative bacterium that harbors the pVA1 plasmid and expresses the *Photorhabdus* insect-related (Pir) binary toxin PirA*^vp^*/PirB*^vp^*. These two toxins, the virulence factors of AHPND, are reported to form a binary complex, destructing the hepatopancreatic tissues of shrimp [8,9]. Upon infection, AHPND-causing bacteria colonize the stomach, and subsequently release the toxic PirA*^vp^* and PirB*^vp^* proteins in the hepatopancreas of shrimp [10]. Since PirA*^vp^* and PirB*^vp^* are both secreted into the digestive tract of shrimp, the proteins can be easily targeted and neutralized by orally administrated compounds or medicated feed [11]. Medicated feed is a common and convenient mode of drug administration in aquaculture [12]. In shrimp farming, oxytetracycline was used to control *Vibrio* infection through medicated feed [13]. Several AHPND measures based on the antimicrobial properties of probiotics [14], prebiotics [15], plant extracts, or essential oils [16] have been applied in shrimp feeds to prevent outbreaks of AHPND. However, the modes of action of these antimicrobial agents are complicated, and not specific in eliminating the causative agents.

The use of peptides as biofunctional ingredients in shrimp aquaculture for disease control has been highlighted. For instance, the application of bioactive peptides to partially replace fish meal in the diet of shrimp was found to promote growth performance and strengthen immunity [17]. Oilseed meals, which are good sources of bioactive peptides [18,19,20], have been shown to contribute to improved shrimp health when used as shrimp feed ingredients [21,22]. Although the aforementioned studies [21,22] did not investigate how oilseed meals specifically affect the incidence of AHPND, the incorporation of peptides derived from *Bacillus subtilis* E20-fermented soybean meal into shrimp feed protected shrimp against AHPND [23]. Hence, the application of oilseed-derived bioactive peptides as toxin-neutralizing agents for the inhibition of AHPND could be a promising anti-AHPND strategy. Research in this area could provide valuable information for the formulation of a novel anti-AHPND shrimp feed fortified with oilseed-derived peptides. With the availability of the crystal structures of PirA*^vp^* and PirB*^vp^* and knowledge of the structural characteristics of the PirA*^vp^*/PirB*^vp^* complex, structure-based drug design, as recommended by [11,24], is a feasible and efficient approach to discover potential anti-AHPND agents. Specifically, in silico screening for inhibitors of PirA*^vp^*/PirB*^vp^* complex formation was proposed to be a promising strategy [24]. Therefore, this study adopted an in silico approach to discover oilseed-derived peptides that can bind to the key regions responsible for PirA*^vp^*/PirB*^vp^* complex formation. The binding of such peptides to the key regions is anticipated to preclude PirA*^vp^*/PirB*^vp^* complex formation. Major proteins of five oilseed meals, namely *Cannabis sativa* (hemp), *Cucurbita maxima* (pumpkin), *Brassica napus* (rape), *Sesamum indicum* (sesame), and *Helianthus annuus* (sunflower) were investigated as sources of PirA*^vp^*- and PirB*^vp^-* binding peptides following tryptic digestion. Screening for non-toxicity, low water solubility, and high thermal stability was also conducted to increase the chance of discovering peptides that can be readily incorporated as shrimp feed ingredients.

## 2. Results and Discussion

### 2.1. In Silico Tryptic Hydrolysis of Oilseed Meal Proteins

To discover peptides that can potentially block the formation of the PirA*^vp^*/PirB*^vp^* toxin complex, we focused on peptides resulting from the in silico trypsin digestion of oilseed proteins. The in vitro trypsin digestion of feedstuffs is comparable to that of in vivo whiteleg shrimp digestion in terms of protein digestibility [25]. Thus, in silico tryptic digestion can be used to simulate the in vivo shrimp digestion of oilseed proteins and predict the peptide fragments that are released in the shrimp digestive tract. Peptides resulting from tryptic digestion also represent peptides that can be fed to the shrimp directly and remain undegraded in the shrimp digestive tract. Either way, such peptides could remain intact in the shrimp digestive tract, and would hence be available for neutralizing the toxicity of the pathogenic *V. parahaemolyticus* strains. In silico trypsin hydrolysis released 809 peptide fragments from 37 oilseed meal proteins (Table 1). Rape and hemp seed proteins released 272 and 247 peptide fragments, respectively, accounting for about a 4.5-fold greater than those from the sunflower proteins (Table 1). Regardless of oilseed type, in silico trypsin hydrolysis generated more peptide fragments from the globulin-type proteins than from the albumin-type. For instance, 93% of the hemp peptides resulting from tryptic hydrolysis was generated from the globulin-type proteins (edestin 1–3 and 7S vicilin-like protein) (Table 1). A similar trend was observed in other studies of the in silico hydrolysis of oilseed proteins [26,27]. Overall, our results suggest that the globulin-type proteins of oilseeds are prominent sources of peptides that could remain intact in the shrimp digestive tract.

### 2.2. Toxicity of Peptides

A bottleneck in the success of drugs used in aquaculture is their toxicity [7]. Peptides to be adopted as a potential anti-AHPND strategy should ideally be non-toxic to shrimp, other aquatic organisms, or human consumers. Therefore, by using the online admetSAR and ToxinPred tools, we screened the 809 peptides liberated from oilseed proteins by in silico hydrolysis for potential toxicity to crustaceans, fish, and humans. Remarkably, all the peptides were predicted as non-toxic to crustaceans (Table 2). Our results suggest that the five oilseed meals appear safe as protein ingredients in shrimp feed. This is in agreement with the report of good survival rate (>87%) in shrimp fed 25–100 g/kg rapeseed meal [28]. In other growth trials, the inclusion of 20–40% sesame meal and 1–10% sunflower cake to shrimp diets also led to good survival rates and health conditions [29,30]. Our results show that 25–35% of the peptides released from the five oilseeds were potentially toxic to fish (Table 2). Our results concur with previous observations of adverse effects in fish fed oilseed-containing diets at high inclusion levels. For instance, fish fed a diet containing 25% sesame meal as a replacement for soybean meal displayed comparable growth performance and survival rate to the control group; but a diet containing 75–100% sesame meal reduced growth performance and survival [31]. Besides a decline in weight gain, the specific growth rate and survival rate of fish was observed when 25–100% of soybean meal was substituted with pumpkin seed meal in fish feed [32]. Our results indicate that although all of the oilseed-derived peptides we investigated in this study were likely non-toxic to shrimp, some of them may be toxic to fish. The drugs used in aquaculture may enter environment, where they contaminate wild seafood [7]. Hence, in this study, only the 580 peptides that were predicted as being non-toxic to fish were considered in our subsequent analysis when searching for potential anti-AHPND peptides. Meanwhile, our ToxinPred analysis revealed that 96–100% of tryptic peptides generated from the oilseed proteins were non-toxic to human. This consideration is important as residual drugs in treated aquatic organisms, including shrimp, can pose health issues to people who consume aquatic food products [7]. Altogether, our toxicity screening narrowed down the initial pool of 809 peptides to a set of 572 oilseed-derived peptides which are potentially non-toxic to shrimp, fish and humans. Such peptides, 463 derived from globulin- and 109 from albumin-type proteins of the five oilseeds, were promising peptides for subsequent search of anti-AHPND agents in this study.

### 2.3. Water Solubility and Thermal Stability of Peptides

In shrimp farming, feed ingredients with high water solubility may be rapidly leached and lost on exposure to water, thus reducing their availability for shrimp ingestion [33]. Thus, in our search for potential anti-AHPND peptides as in-feed medication for shrimp, we further screened the 572 non-toxic oilseed-derived peptides for water solubility. This revealed 59 low-water-solubility peptides (data not shown), each made of 25% to 100% hydrophobic amino acids (e.g., Ala, Val, and Phe). This is in agreement with the finding that the nonpolar portion of peptides accounts for their low solubility in aqueous solutions [34]. In both feed processing and aquafarming, highly water-soluble compounds are prone to leaching. For instance, water-soluble crystalline amino acids were reported to leach out rapidly from shrimp feed, compromising the nutritional quality of shrimp diets [33]. Another study also pointed out that the leaching of water-soluble protein hydrolysates or peptides from feed may weaken the responses of fish to diets supplemented with such protein hydrolysates or peptides [35]. To minimize the potential issue of leaching, only the 59 low-water-solubility oilseed peptides were selected for further analysis in our search for in-feed anti-AHPND peptides. The 59 peptides included some repetitive sequences. For example, two identical sequences (i.e., GVLYK) could be found among the non-toxic hemp-derived peptides. By excluding the repetitive sequences, the 59 low-water-solubility peptides were consolidated into a set of 49 peptides with unique sequences, as shown in Table 3.

Thermal treatment is an important part of aquaculture feed production [36]. Hence, anti-AHPND peptides to be incorporated into shrimp feed should be heat-stable. In this study, the thermal stability of the 49 low-water-solubility oilseed peptides were predicted based on their aliphatic index and compared with lantibiotic nisin-A (Table 3). The aliphatic index, computed based on the percentage of aliphatic amino acids (Ala, Ile, Leu, and Val), is correlated with thermal stability to proteins [37]. Based on their aliphatic indices, 24 of the 49 peptides were likely thermally more stable than nisin-A (Table 3). This is notable as nisin-A is a heat-stable peptide that retains its antimicrobial activity after thermal treatment at 121 °C for 15 min; it has also been used as food preservative for decades [38]. The thermal stability predicted for the 24 oilseed-derived peptides could be attributed, at least in part, to a relatively high percentage of aliphatic amino acids in their sequences. For instance, PI and PVV, the sesame- and hemp-derived peptides predicted as having the highest aliphatic indices, are composed of 50% and 67% of aliphatic amino acids, respectively (Table 3). Taken together, our analysis led to 24 oilseed-derived peptides with favorable properties namely non-toxicity, low water solubility, and high thermal stability.

### 2.4. Molecular Docking Analysis

**Figure 1:**
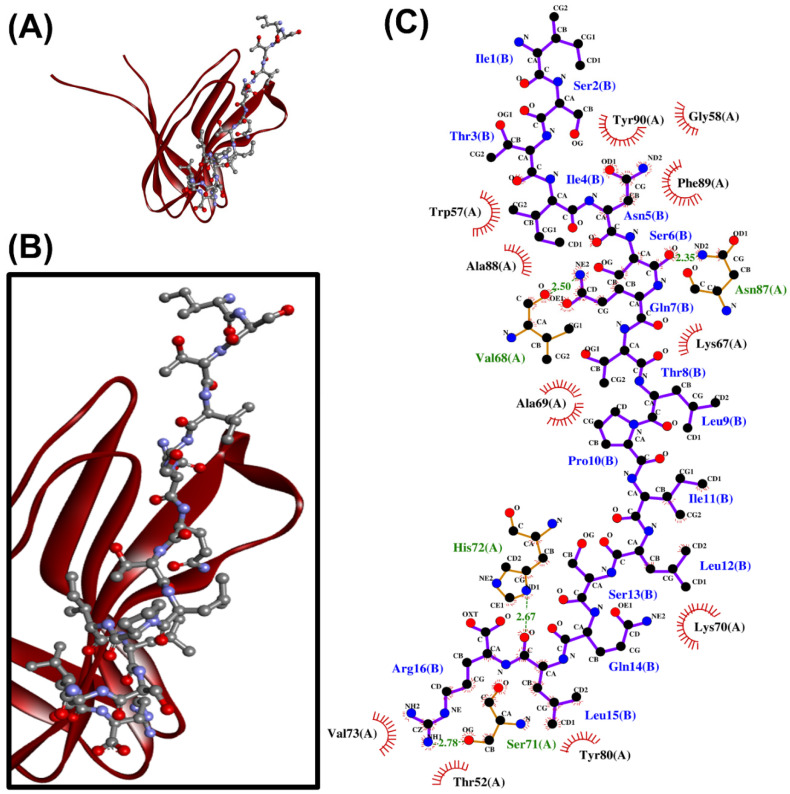
The ISTINSQTLPILSQLR-PirA*^vp^* docked model presented in 3D (**A**,**B**) and 2D (**C**) diagrams. In (**A**,**B**), PirA*^vp^* is shown in the cartoon in maroon; ISTINSQTLPILSQLR is presented in a ball-and-stick style. In the 2D diagram (**C**), the hydrophobic bonds and hydrogen bonds are displayed in red spoked arcs and green dashed lines, respectively.

Interception of the complex formation between PirA*^vp^* and PirB*^vp^* was recommended as a promising anti-AHPND strategy [24]. To discover peptides that could preclude the formation of PirA*^vp^*/PirB*^vp^* complex, the 24 aforementioned peptides were each docked onto PirA*^vp^* and PirB*^vp^*. When docking onto PirA*^vp^,* 18 peptides produced similar or more negative docking scores (−144.315 to −194.881) than the six PirB*^vp^* regions (−142.536 to −174.899) which we used for comparison (Table 4). Hence, when compared with PirB*^vp^*, the 18 peptides potentially bound more stably to PirA*^vp^*. This implies the potential of the 18 peptides to intercept the binding between PirA*^vp^*-PirB*^vp^*. Intermolecular interactions between the 18 peptides and PirA*^vp^* were analyzed by using LigPlot+. The 18 peptides could each form interactions with 4–10 residues within the two PirB*^vp^*-binding regions of PirA*^vp^* (Table 4 and Table S1). For example, sunflower-derived VIQNLPNQCDLEVQQCTTCTG could bind to one residue (Val23) in the 15-WTVEPNGGVTEVDSKHTPIIPEVGRS-40 region, and to five residues (Thr52, Gln54, Ser71, Gln75, and Arg76) in the 52-TIQYQWGAPFMAGGWKVAKSHVVQRDET-79 region of PirA*^vp^.* Overall, most of the predicted peptide-PirA*^vp^* binding involved the 52-TIQYQWGAPFMAGGWKVAKSHVVQRDET-79 region of PirA*^vp^.* This region was also responsible for most of the predicted binding between the six PirB*^vp^* regions and PirA*^vp^* (Supplementary Materials Table S1). Overall, hydrophobic interactions formed the majority of the peptide-PirA*^vp^* interactions. A similar trend was observed when the six PirB*^vp^* regions were docked onto PirA*^vp^* (Table 4). In particular, sesame-derived ISTINSQTLPILSQLR stood out among the 18 peptides due to its largest number of interactions with residues in 52-TIQYQWGAPFMAGGWKVAKSHVVQRDET-79, including Lys67 and Lys70 (Table 4 and Table S1). The two Lys residues were found to be localized in the dimeric interface between PirA*^vp^* and PirB*^vp^*. The binding of peptides to the two Lys residues may block the cross-linking between PirA*^vp^* with PirB*^vp^* [24]. A graphical representation of the intermolecular interactions between ISTINSQTLPILSQLR and PirA*^vp^* is shown in Figure 1.

**Figure 2:**
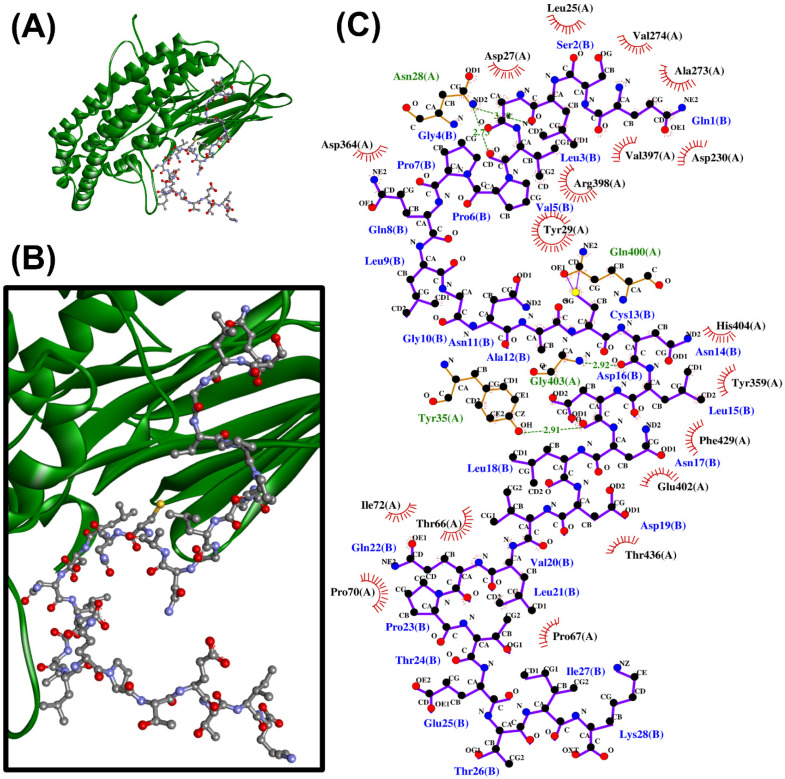
The QSLGVPPQLGNACNLDNLDVLQPTETIK-PirB*^vp^* docked model presented in 3D (**A**,**B**) and 2D (**C**) diagrams. In (**A**,**B**), PirB*^vp^* is shown in the cartoon in green; QSLGVPPQLGNACNLDNLDVLQPTETIK is presented in a ball-and-stick style. In the 2D diagram (**C**), the hydrophobic bonds, hydrogen bonds, and external bonds are displayed in red spoked arcs, green dashed lines, and purple lines, respectively.

**Figure 3:**
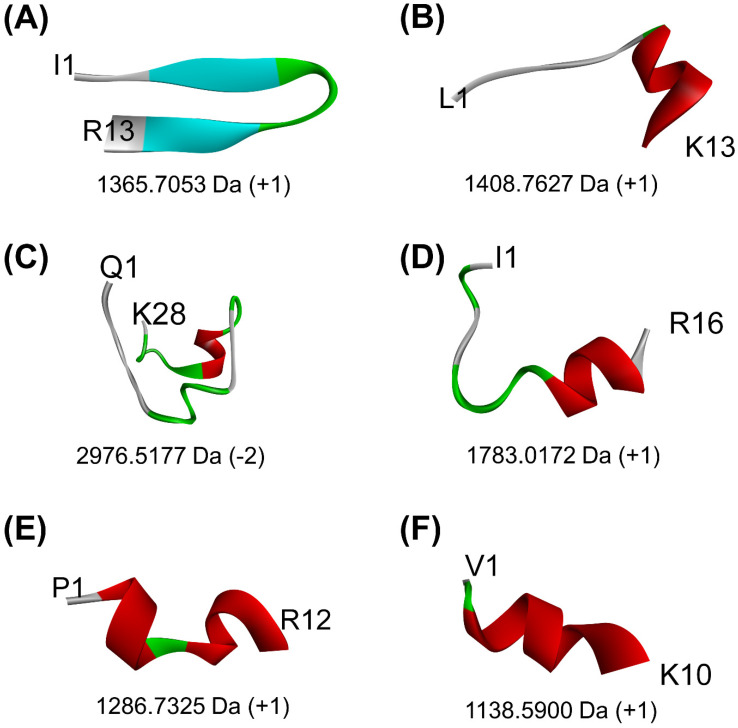
The 3D structures of six peptides predicted to bind to the key regions of both PirA*^vp^* and PirB*^vp^* that are involved in PirA*^vp^*/PirB*^vp^* complex formation: (**A**) ISYVVQGMGISGR, (**B**) LTFVVHGHALMGK, (**C**) QSLGVPPQLGNACNLDNLDVLQPTETIK, (**D**) ISTINSQTLPILSQLR, (**E**) PQFLVGASSILR, and (**F**) VQVVNHMGQK. The alpha-helical elements are shown in maroon, whereas the beta-strands are shown in light blue. Only the N- and C-terminal residues of each peptide are indicated. Values below each structure represent the molecular mass and the net charge of the peptide, as computed by PepDraw.

When the 24 non-toxic, low-water-solubility, and thermally-stable oilseed peptides were docked onto PirB*^vp^,* only six produced more negative docking scores, suggesting more stable binding, than the PirB*^vp^*-binding region 15-WTVEPNGGVTEVDSKHTPIIPEVGRS-40 of PirA*^vp^*. By contrast, all of the 24 peptides could not form a more stable binding to PirB*^vp^* when compared with the PirA*^vp^* region 52-TIQYQWGAPFMAGGWKVAKSHVVQRDET-79 (Table 5). A LigPlot+ analysis also revealed that the six best-scored peptides could bind to 2–4 residues within only two of the six PirA*^vp^*-binding regions of PirB*^vp^*, namely 386-FVVGENSGKPSVRLQL-401 and 426-YELFHPDEF-434. For instance, rape-derived QSLGVPPQLGNACNLDNLDVLQPTETIK, predicted to form the largest number of interactions with PirB*^vp^*, could bind to Val397, Arg398, and Gln400 in the 386-FVVGENSGKPSVRLQL-401 region, and could bind to Phe429 in the 426-YELFHPDEF-434 region of PirB*^vp^*. Figure 2 shows the intermolecular interactions contributing to QSLGVPPQLGNACNLDNLDVLQPTETIK-PirB*^vp^* binding. Similarly, limited interactions between the two PirA*^vp^* regions 15-WTVEPNGGVTEVDSKHTPIIPEVGRS-40 and 52-TIQYQWGAPFMAGGWKVAKSHVVQRDET-79 with PirB*^vp^* were also observed (Table 5 and Table S2). Interestingly, the six oilseed peptides having predicted interactions with PirB*^vp^* (Table 5) were also among the 18 best-scoring PirA*^vp^*-binding peptides presented in Table 4. The six peptides ranged between 1139 Da and 2977 Da in mass, and 10–28 residues in length, mostly having a net charge of +1. Modeling with PEP-FOLD 3.5 revealed the 3D structures of all the peptides except the rape-derived ISYVVQGMGISGR to be comprised of helical elements (Figure 3). The six peptides were derived from globulin-type proteins from rape, sesame, and hemp. Our findings suggest that the globulin-type proteins of these three seeds are potential sources of peptides that can bind to both PirA*^vp^* and PirB*^vp^*. As observed in oilseed peptide-PirA*^vp^* interactions (Table 4), hydrophobic interactions contributed to the majority of the interactions between the six peptides and PirB*^vp^* (Table 5). Taken together, our results suggest that hydrophobic interactions are critical in facilitating stable binding between the six oilseed peptides and the proteins of both PirA*^vp^* and PirB*^vp^*.

The virulence factors of AHPND have been shown to be both PirA*^vp^* and PirB*^vp^* [9,39]. Hence, targeting only one of the proteins may not result in full protection against AHPND. For example, the binding of the chicken egg yolk immunoglobulin (IgY) specific for a recombinant PirB*^vp^*-like protein (anti-PirB*^vp^*-IgY) to the PirB*^vp^* toxin still allowed AHPND infection to progress, resulting in a 14% survival rate of AHPND-challenged shrimp [40]. On the other hand, the binding of the IgY specific for the recombinant PirA*^vp^*-like protein (anti-PirA*^vp^*-IgY) to PirA*^vp^* gave rise to only an 86% survival rate in AHPND-challenged shrimp [40]. Therefore, we speculate that dual-target oilseed peptides that can bind to both PirA*^vp^* and PirB*^vp^* may be a relatively promising approach for intercepting PirA*^vp^*/PirB*^vp^* complex formation, thus mitigating AHPND occurrence in shrimp. Based on our results, the six oilseed peptides that can bind to both PirA*^vp^* and PirB*^vp^* (ISYVVQGMGISGR, LTFVVHGHALMGK, QSLGVPPQLGNACNLDNLDVLQPTETIK, ISTINSQTLPILSQLR, PQFLVGASSILR, and VQVVNHMGQK) deserve attention in future research. In this theoretical investigation, we considered peptides undegraded by trypsin to be bioavailable in the shrimp digestive tract. Considering the complexity of factors governing bioavailability, investigation into the in vivo availability of the six aforementioned peptides should be the first priority in future research. Next, testing of the in vivo effectiveness of these peptides individually and in combination in live shrimp, delivered as ingredients incorporated into shrimp feed, would be of great interest. Besides validating the properties predicted in this study (absence of toxicity, low water solubility, and thermal stability) and confirming the anti-AHPND potency of the peptides, such studies may also reveal whether the peptides could function synergistically. An interesting recent development is the novel discovery by Almanza-Martínez et al. [41] of an α-amylase-like protein serving as a binding receptor to PirB*^vp^* in the digestive tract tissue of the whiteleg shrimp. The specific role of the α-amylase-like protein in the mechanism of action of PirB*^vp^* remains to be established, as are the residues participating in the α-amylase-PirB*^vp^* binding interface. Once such information has been elucidated, it would be interesting to investigate, through molecular docking and/or in vitro experiments, whether the oilseed peptides reported in this study can also intercept the α-amylase-PirB*^vp^* interaction. Meanwhile, the potential off-target interactions of the six selected oilseed peptides should also be addressed to ensure that they do not disrupt other normal biological processes, causing adverse side-effects in the shrimp.

## 3. Materials and Methods

### 3.1. In Silico Hydrolysis of Oilseed Meal Proteins

A total of 37 major proteins found in five oilseed meals, namely those of hemp [42,43], pumpkin [44], rape [45], sesame [20], and sunflower [46] were chosen for this study. The major proteins chosen can be classified into two types, globulins and albumins, which account for more than 78% of seed protein constituents [43,44,45,46,47]. The sequence of each protein was downloaded from the UniProt Knowledgebase (https://www.uniprot.org/) [48] on 5 May 2021. All 37 proteins were hydrolyzed by trypsin (EC 3.4.21.4) using the “Enzyme(s) action” tool on the BIOPEP-UWM bioactive peptides database (http://www.uwm.edu.pl/biochemia/index.php/en/biopep) [49] on 23 May 2021.

### 3.2. Toxicity of Peptides

Aquatic toxicity of peptides towards crustaceans and fish was analyzed by using admetSAR (http://lmmd.ecust.edu.cn/admetsar2) [50]. Peptide sequences were converted into the molecular input line entry system (SMILES) format using BIOPEP-UWM (http://www.uwm.edu.pl/biochemia/index.php/en/biopep) [49] before analysis with admetSAR. Toxicity of peptides to humans was predicted with ToxinPred (https://webs.iiitd.edu.in/raghava/toxinpred/multi_submit.php) [51]. Support vector machine (SVM) and the default SVM threshold of 0.0 were chosen for ToxinPred toxicity prediction. Peptides with an SVM score < 0.0 were predicted as non-toxic. The aforementioned analyses were performed between 25 May and 31 August 2021. 

### 3.3. Water Solubility and Thermal Stability of Peptides

Water solubility of peptides were analyzed using an online peptide property calculator (https://pepcalc.com/). Aliphatic index, an indicator of thermal stability, was predicted by using the Peptides Package in R (https://rdrr.io/snippets/) [37]. The two online tools were accessed between 26 to 31 August 2021. 

### 3.4. Molecular Docking Analysis

Oilseed peptides predicted as non-toxic to crustaceans, fish and humans, with low water solubility and high thermal stability, were chosen for molecular docking analysis. The two target proteins from *V. parahaemolyticus*, namely PirA*^vp^* (Protein Data Bank (PDB) code: 3X0T) and PirB*^vp^* (PDB code: 3X0U) [9] were used as receptors. The docking of peptides onto chain A of PirA*^vp^* and of PirB*^vp^* was performed by submitting PDB ID: Chain ID information and peptide sequences to HPEPDOCK (http://huanglab.phys.hust.edu.cn/hpepdock/) [52]. For comparison, two PirA*^vp^* regions (15-WTVEPNGGVTEVDSKHTPIIPEVGRS-40 and 52-TIQYQWGAPFMAGGWKVAKSHVVQRDET-79), and six PirB*^vp^* regions (214-WADNDSYNNANQD-226, 290-DEIPQPLKPNM-300, 322-YNRVGRLKL-330, 386-FVVGENSGKPSVRLQL-401, 409-MLADQEGSDKVAA-421, and 426-YELFHPDEF-434) reported to be involved in PirA*^vp^*/PirB*^vp^* complex formation [24], were used. These sequences were docked onto each corresponding target protein as described above. All docking analyses were carried out between 27 May 2021 and 31 August 2021. The best (most negative) docking score of each peptide was tabulated. Three-dimensional (3D) structures of the protein-peptide docked models were visualized by using BIOVIA Discovery Studio Visualizer (BIOVIA, Dassault Systèmes, BIOVIA Discovery Studio Visualizer, Version 20.1.0.192, San Diego: Dassault Systèmes, 2020). Two-dimensional (2D) protein-peptide interaction diagrams were generated with LigPlot+ v.2.2 [53,54], through which intermolecular interactions were visualized and analyzed. PEP-FOLD 3.5 (https://bioserv.rpbs.univ-paris-diderot.fr/services/PEP-FOLD3/) [55,56,57] was used to model the 3D structures of selected oilseed peptides, setting the number of simulations to 200 and with model sorting based on sOPEP energy. PepDraw (http://pepdraw.com/) was used to compute the molecular masses and net charges of the selected peptides. PEP-FOLD 3.5 and PepDraw were accessed on 1 September 2021.

## 4. Conclusions

Our in silico study found the globulin-type proteins of the oilseeds investigated to be promising sources of PirA*^vp^*- and PirB*^vp^-* binding peptides. Upon selection for non-toxicity against crustaceans, fish, and humans, as well as thermal stability and low water solubility, six peptides that could bind to both PirA*^vp^* and PirB*^vp^* with stability comparable or superior to the binding between the key regions in the native PirA*^vp^*/PirB*^vp^* complex, were identified. These six peptides could potentially play a role in intercepting the formation of the toxic PirA*^vp^*/PirB*^vp^* complex, thus serving as promising anti-AHPND agents. The findings of this study can be used to guide the future discovery and development of anti-AHPND peptides from oilseed proteins.

## Figures and Tables

**Table 1:** Numbers of peptide fragments released from major oilseed proteins by in silico trypsin hydrolysis.

Source	Protein	UniProt Accession	Type *	Number of Residues	Number of Peptides
Hemp	Albumin	A0A219D1L6	A	119	18
	Edestin 1	A0A090DLH8	G	488	62
	Edestin 2	A0A090DLI7	G	467	56
	Edestin 3	A0A219D3H6	G	468	54
	7S vicilin-like protein	A0A219D1T7	G	472	57
Pumpkin	2S albumin large chain	Q39649	A	67	13
	11S globulin delta chain	P13744	G	184	24
	11S globulin gamma chain	P13744	G	275	32
Rape	Cruciferin BnC1 subunit alpha	P33523	G	277	24
	Cruciferin BnC1 subunit beta	P33523	G	190	16
	Cruciferin BnC2 subunit alpha	P33524	G	283	22
	Cruciferin BnC2 subunit beta	P33524	G	190	19
	Cruciferin CRU1 alpha chain	P33525	G	296	22
	Cruciferin CRU1 beta chain	P33525	G	190	17
	Cruciferin CRU4 alpha chain	P33522	G	254	20
	Cruciferin CRU4 beta chain	P33522	G	189	17
	Cruciferin subunit alpha	P11090	G	275	24
	Cruciferin subunit beta	P11090	G	190	16
	Napin-1A small chain	P24565	A	31	5
	Napin-1A large chain	P24565	A	79	8
	Napin-2 small chain	P01090	A	36	6
	Napin-2 large chain	P01090	A	81	9
	Napin-3 small chain	P80208	A	37	6
	Napin-3 large chain	P80208	A	88	9
	Napin-B small chain	P27740	A	36	6
	Napin-B large chain	P27740	A	84	10
	Napin embryo-specific small chain	P09893	A	38	6
	Napin embryo-specific large chain	P09893	A	89	10
Sesame	2S seed storage protein 1 small subunit	Q9XHP1	A	30	6
	2S seed storage protein 1 large subunit	Q9XHP1	A	70	10
	11S globulin isoform 3	Q2XSW7	G	468	54
	11S globulin isoform 4	Q2XSW6	G	449	46
	11S globulin seed storage protein 2 acidic chain	Q9XHP0	G	256	31
	11S globulin seed storage protein 2 basic chain	Q9XHP0	G	182	18
Sunflower	2S seed storage protein	P15461	A	134	15
	11S globulin seed storage protein G3 acidic chain	P19084	G	285	22
	11S globulin seed storage protein G3 basic chain	P19084	G	188	19

* G, globulin-type; A, albumin-type.

**Table 2:** In silico prediction of crustacean, fish and human toxicity of oilseed-derived peptides.

Source	Protein Type *	Total Number of Peptides	Number of Peptides Predicted as Non-Toxic (NT) and Toxic (T) against Different Organisms					
			Crustacean		Fish		Human	
			NT	T	NT	T	NT	T
Hemp	G	229	229	0	170	59	229	0
	A	18	18	0	16	2	17	1
Pumpkin	G	56	56	0	40	16	56	0
	A	13	13	0	12	1	12	1
Rape	G	197	197	0	115	82	194	0
	A	75	75	0	61	14	70	5
Sesame	G	149	149	0	109	40	149	0
	A	16	16	0	15	1	14	2
Sunflower	G	41	41	0	29	12	40	0
	A	15	15	0	13	2	14	1
Total		809	809	0	580	229	795	10

* G, globulin-type; A, albumin-type.

**Table 3:** Aliphatic indices of 49 oilseed-derived peptides predicted to be non-toxic and having low water solubility, in comparison with reference peptide lantibiotic nisin-A.

Source	Peptide	Aliphatic Index
Sesame	PI	195
	GLIVMAR	167
	GHIITVAR	146
	ISTINSQTLPILSQLR	146
	IQVVGHK	139
	GVLYR	136
	GLQVISPPLQR	133
	VASA	123
	QEQFQCAGIVAMR	68
	MTFVR	58
	QTFHNIFR	49
	YWQSLQQHQQHR	33
	GQHQFGNVFR	29
	TGGYA	20
	HCMQWMR	0
	GSTWQQGQCR	0
Hemp	PVV	193
	GVLYK	136
	PQFLVGASSILR	130
	QGQIVTVPQNHAVVK	110
	ESVILPTSAASPPVK	104
	LGNLTSYQR	87
	VQVVNHMGQK	87
	ATA	67
	NIPSMCGMQPR	35
	TTWSWR	0
	WQSQCQFQR	0
Sunflower	GHIVNVGQDLQIVR	146
	VIQNLPNQCDLEVQQCTTCTG	83
	WVSFK	58
	GGWSN	0
Rape	LTFVVHGHALMGK	112
	QSLGVPPQLGNACNLDNLDVLQPTETIK	108
	ISYVVQGMGISGR	107
	TNANAQINTLAGR	83
	NLPNVCNMK	76
	TNANAMVSTLAGR	75
	CSGVSFVR	73
	ACQQWIR	70
	ACQQWLHK	61
	ATSQQFQWIEFK	41
	QQQGQQMQGQQMQQVISR	38
	VQGQHGPFQSTR	24
	QAMQSGGG	13
	QAMQSGSG	13
	QAMQPGGGSG	10
	TMPG	0
	TMPGPS	0
	TMPGPSY	0
Lantibiotic nisin-A	ITSISLCTPGCKTGALMGCNMKTATCHCSIHVSK	72

**Table 4:** Docking scores for interactions between the 18 best-scored oilseed peptides and PirA*^vp^*, with details on the PirA*^vp^* residues involved in different types of interactions.

Source	Peptide	Docking Score	Interaction with PirA*^vp^* Residues *			
			Hydrogen Bond	Hydrophobic Interaction	Salt Bridge	External Bond
Rape	LTFVVHGHALMGK	−194.881	Asn87	**Trp57**, **Gly58**, **Pro60**, **Ala63**, **Ala69**, **Lys70**, Tyr80, Gln83, Pro85, Asn87, Ala88, Phe89, Tyr90	-	-
	ISYVVQGMGISGR	−174.661	Tyr80(2)	**Trp57**, **Gly58**, **Ala59**, **Pro60**, **Val68**, **Ala69**, **Lys70**, **Ser71**, **His72**, Tyr80, His81, Leu82, Gln83, Pro85, Asn87, Phe89, Tyr90	-	-
	QSLGVPPQLGNACNLDNLDVLQPTETIK	−168.015	**Gln54**, **Trp57**, Ser71(2), Tyr80, Asn87	**Thr52**, **Gln54**, **Trp57**, **Val68**, **Ala69**, **Lys70**, **Ser71**, **His72**, Tyr80, His81, Pro85, Asn87, Phe89, Asn99	-	Asn99(3)
	TNANAMVSTLAGR	−156.242	**Ala69**, **Ser71**	**Pro35**, **Trp57**, **Val68**, **Ala69**, **Lys70**, **Ser71**, **His72**, Asn87, Tyr80, Ala88, Tyr90	-	-
	TNANAQINTLAGR	−151.751	**Ala69**, **His72**, Tyr80, Asn87(2)	**Trp57**, **Ala59**, **Pro60**, **Val68**, **Ala69**, **Lys70**, **Ser71**, **His72**, Tyr80, Pro85, Asn87, Phe89, Tyr90	-	-
	NLPNVCNMK	−147.564	**Lys29**, **Arg39**(3), Arg84	Tyr11, **Lys29**, **Gly38**, **Arg39**, **Ser40**, Arg84, Asp86, His111, Leu112, Glu113, His114, His115	Asp86	Arg84
	CSGVSFVR	−146.934	Asn87	**Trp57**, **Gly58**, **Ala59**, **Pro60**, **Ala69**, **Lys70**, Pro85, Asp86, Asn87, Ala88, Phe89, Tyr90	-	Asp86
Sesame	ISTINSQTLPILSQLR	−178.623	**Val68**, **Ser71**, **His72**, Asn87	**Thr52**, **Trp57**, **Gly58**, **Lys67**, **Val68**, **Ala69**, **Lys70**, **Ser71**, **His72**, **Val73**, Tyr80, Asn87, Ala88, Phe89, Tyr90	-	-
	GLQVISPPLQR	−171.949	Asp10, **Lys29**, **Arg39**, Glu113	Asp10, His13, **Trp15**, **Asp27**, **Ser28**, **Lys29**, **Gly38**, **Arg39**, **Ser40**, Arg84, Gln92, Tyr110, His111, Leu112, Glu113, His114, His115	Asp10	Arg84
	GHIITVAR	−151.394	**Gly58**, **Ser71**	**Trp57**, **Gly58**, **Ala59**, **Pro60**, **Val68**, **Ala69**, **Ser71**, **His72**, Asn87, Phe89, Tyr90	-	-
	GLIVMAR	−144.315	Arg84(3)	**Lys29**, **Val37**, **Gly38**, **Arg39**, Gln83, Arg84, Pro85, Asp86, His111, Leu112, Glu113, His114, His115	Asp86(3)	Glu113
Hemp	QGQIVTVPQNHAVVK	−177.911	Tyr80, Asn87, Tyr90	**Trp57**, **Pro60**, **Ala69**, **Lys70**, **Ser71**, **His72**, Tyr80, Leu82, Asn87, Ala88, Phe89, Tyr90	-	**Ala69**
	PQFLVGASSILR	−175.973	**Trp57**, **Ser71**, Asn87	**Trp57**, **Lys67**, **Val68**, **Lys70**, **Ser71**, **His72**, Tyr80, His81, Leu82, Gln83, Asn87, Ala88, Phe89	-	His81
	LGNLTSYQR	−165.451	**Trp57**, **Ser71**, Tyr80, Arg84, Asn87	**Trp57**, **Val68**, **Ala69**, **Lys70**, **Ser71**, **His72**, Tyr80, Leu82, Gln83, Asp86, Asn87, Ala88, Tyr90	Asp86(5)	-
	VQVVNHMGQK	−155.517	**Ala69**, **Ser71**, Ala88	**Trp57**, **Ala59**, **Pro60**, **Val68**, **Ala69**, **Lys70**, Asn87, Ala88, Phe89, Tyr90	-	-
	ESVILPTSAASPPVK	−155.494	**Lys70**, Asn87	**Pro35**, **Trp57**, **Gly58**, **Ala59**, **Pro60**, **Val68**, **Ala69**, **Lys70**, **Ser71**, Tyr80, His81, Leu82, Pro85, Asn87, Ala88, Phe89, Tyr90	-	-
Sunflower	GHIVNVGQDLQIVR	−175.043	**Ala69**, **His72**, Asn87	**Trp57**, **Pro60**, **Val68**, **Ala69**, **Lys70**, **His72**, Tyr80, Asn87, Ala88, Tyr90	**Lys70**	-
	VIQNLPNQCDLEVQQCTTCTG	−172.423	Arg48, **Ser71**(2), **Gln75**, **Arg76**	**Val23**, Arg48, Gly49, Glu50, **Thr52**, **Gln54**, **Ser71**, **Gln75**, **Arg76**, Ile97, Asn99, Gly100, Asn101	Arg48(2)	**Thr52**
PirB*^vp^*	YNRVGRLKL	−174.899	**Trp57**, **Val68**, Asn87, Ala88	**Trp57**, **Val68**, **Ala69**, **Lys70**, **Ser71**, **His72**, Tyr80, Leu82, Pro85, Asn87, Ala88, Phe89	-	-
	WADNDSYNNANQD	−170.618	**His72**, Asn87, Ala88	**Pro35**, **Ile53**, **Trp57**, **Ala69**, **Lys70**, **Ser71**, **His72**, Tyr80, Leu82, Pro85, Asn87, Ala88, Phe89	**Lys70**	Leu82(2)
	FVVGENSGKPSVRLQL	−167.625	**Ser28**, **Gly38**, Gln83, His111, Leu112	**Ser28**, **Lys29**, **His30**, **Thr31**, **Ile33**, **Glu36**, **Val37**, **Gly38**, **Arg39**, **Ser40**, His81, Gln83, Arg84, His111, Leu112, Glu113	**Glu36**	**Ser28**, **Ile33**
	YELFHPDEF	−159.953	**Ser71**(3), Tyr80	**Pro35**, **Trp57**, **Lys67**, **Val68**, **Ala69**, **Lys70**, **Ser71**, **His72**, Tyr80, Asn87, Ala88, Phe89, Tyr90	-	**Val68**
	DEIPQPLKPNM	−148.916	Tyr80, Asn87	**Trp57**, **Gly58**, **Ala69**, **Lys70**, Tyr80, Pro85, Asn87, Ala88, Phe89, Tyr90	-	-
	MLADQEGSDKVAA	−142.536	**Lys70**, **Ser71**, Asn87(3)	**Trp57**, **Gly58**, **Ala59**, **Pro60**, **Val68**, **Ala69**, **Lys70**, **Ser71**, **His72**, **Val73**, Asn87, Phe89, Tyr90	-	**Ser71**

* Residues within two PirA*^vp^* regions (15-WTVEPNGGVTEVDSKHTPIIPEVGRS-40 and 52-TIQYQWGAPFMAGGWKVAKSHVVQRDET-79) that were previously reported to bind to PirB*^vp^* to form a toxic complex are marked in boldface type. Number in brackets indicates the number of interactions. Six PirB*^vp^* regions (322-YNRVGRLKL-330, 214-WADNDSYNNANQD-226, 386-FVVGENSGKPSVRLQL-401, 426-YELFHPDEF-434, 290-DEIPQPLKPNM-300, and 409-MLADQEGSDKVAA-421) that were previously reported to bind to PirA*^vp^* to form a toxic complex were analyzed for comparison.

**Table 5:** Docking scores for interactions between six best-scored oilseed peptides and PirB*^vp^*, with details on the PirB*^vp^* residues involved in different types of interactions.

Source	Peptide	Docking Score	Interaction with PirB*^vp^* Residues *		
			Hydrogen Bond	Hydrophobic Interaction	External Bond
Rape	ISYVVQGMGISGR	−209.710	Tyr35, Glu73, Tyr359	Tyr35, Ala36, Ala39, Met40, Phe43, Ile48, Pro49, Asn60, Ile61, Pro64, Asp71, Ile72, Glu73, Gln78, Tyr359, Lys361, **Gln400**, GLn402, **Phe429**, **Pro431**, Thr436	-
	LTFVVHGHALMGK	−208.710	Gly53, Asn60	Tyr35, Ala39, Met40, Tyr50, Ala51, Gly52, Ser56, Thr57, Asn60, Asn114, Glu118, Tyr359, **Phe429**, **Pro431**, **Phe434**, Gly435, Thr436	-
	QSLGVPPQLGNACNLDNLDVLQPTETIK	−201.070	Asn28(2), Tyr35, Gly403	Leu25, Asp27, Asn28, Tyr29, Tyr35, Thr66, Pro67, Pro70, Ile72, Asp230, Ala273, Val274, Tyr359, Asp364, **Val397**, **Arg398**, **Gln400**, Glu402, Gly403, His404, **Phe429**, Thr436	**Gln400**(2)
Sesame	ISTINSQTLPILSQLR	−198.924	Arg21, Tyr29, Tyr359, Ser381, **Arg398**(2)	Arg21, Tyr29, Tyr35, Met40, Phe43, Ile48, Gln84, Asp85, Glu89, Tyr359, Lys361, Tyr362, Ser381, **Arg398**, **Gln400**, Glu402, Thr436	-
Hemp	PQFLVGASSILR	−198.337	Tyr359, Lys361, **Phe434**	Tyr35, Ala36, Ala39, Met40, Ile48, Asn60, Ile61, Pro64, Asn65, Thr66, Ile72, Tyr359, Lys361, Tyr362, Ser381, Asp383, Gly403, **Phe429**, **Phe434**, Gly435, Thr436	Tyr35
	VQVVNHMGQK	−196.671	-	Tyr35, Ala39, Met40, Phe43, Ile48, Pro49, Tyr50, Ala51, Thr57, Asn60, Ile61, Asn65, Thr66, Pro67, Tyr359, Lys361, Gly403, His404, **Phe429**, **Pro431**, Thr436	Asn65
PirA*^vp^*	TIQYQWGAPFMAGGWKVAKSHVVQRDET	−219.311	Asn28, Asn65	Asp27, Tyr29, Glu30, Val31, Tyr35, Met40, Ile48, Tyr50, Ala51, Thr57, Asn60, Pro64, Asn65, Thr66, Gln78, Asp81, Arg82, Gln84, Asp85, Tyr362, **Ser396**, **Val397**, **Arg398**, Glu402	**Val397**
	WTVEPNGGVTEVDSKHTPIIPEVGRS	−191.468	Tyr35, **Ser396**	Asp27, Asn28, Tyr29, Val31, Tyr35, Ala36, Ile72, Gln78, Asp81, Asp364, **Pro395**, **Ser396**, **Val397**, **Arg398**, **Phe429**	-

* Residues within six PirB*^vp^* regions (214-WADNDSYNNANQD-226, 290-DEIPQPLKPNM-300, 322-YNRVGRLKL-330, 386-FVVGENSGKPSVRLQL-401, 409-MLADQEGSDKVAA-421, and 426-YELFHPDEF-434) that were previously reported to bind to PirA*^vp^* to form a toxic complex are marked in boldface type. Number in brackets indicates the number of interactions. Two PirA*^vp^* regions (52-TIQYQWGAPFMAGGWKVAKSHVVQRDET-79 and 15-WTVEPNGGVTEVDSKHTPIIPEVGRS-40) that were previously reported to bind to PirB*^vp^* to form a toxic complex were analyzed for comparison.

## Data Availability

The data presented in this study are available on request from the corresponding author.

## Supplementary Materials

The following are available online at https://www.mdpi.com/article/10.3390/antibiotics10101211/s1, Table S1: Number of residues in the two PirB*^vp^*-binding regions of PirA*^vp^* protein that are involved in interactions with 18 chosen oilseed peptides, Table S2: Number of residues in the six PirA*^vp^*-binding regions of PirB*^vp^* protein that are involved in interactions with six chosen oilseed peptides.
