# Supplementary material for: Targeting PirAvp and PirBvp Toxins of Vibrio parahaemolyticus with Oilseed Peptides: An In Silico Approach
Source: Antibiotics (Basel). 2021 Oct 5;10(10):1211. doi: 10.3390/antibiotics10101211 (PMC8532646; doi:10.3390/antibiotics10101211)
Supplement: Supplementary file 1 [file antibiotics-10-01211-s001.zip › antibiotics-1389663-supplementary.pdf]

Supplementary Materials

Table S1: Number of residues in the two PirB^{vp}-binding regions of PirA^{vp} protein that are involved in interactions with 18 chosen oilseed peptides.

Source	Peptide	PirB^{vp}-Binding Region of PirA^{vp} Protein		Total Number of Residues
		15-WTVEPNGGVTE VDSKHTPIIPEVG RS-40	52-TIQYQWGAPF MAGGWKVAK SHVVQRDET-79	
Rape	LTFVVHGHALMGK	0	6	6
	ISYVVQGMGISGR	0	9	9
	QSLGVPPQLGNACNLDNLDVL QPTETIK	0	8	8
	TNANAMVSTLAGR	1	6	7
	TNANAQINTLAGR	0	8	8
	NLPNVCNMK	4	0	4
	CSGVSFVR	0	6	6
Sesame	ISTINSQTLPILSQLR	0	10	10
	GLQVISPPLQR	7	0	7
	GHIITVAR	0	8	8
	GLIVMAR	4	0	4
Hemp	QGQIVTVPQNHAVVK	0	6	6
	PQFLVGASSILR	0	6	6
	LGNLTSYQR	0	6	6
	VQVVNHMGQK	0	7	7
	ESVILPTSAASPPVK	1	8	9
Sunflower	GHIVNVGQDLQIVR	0	6	6
	VIQNLPNQCDLEVQQCTTCTG	1	5	6
PirB^{vp}	YNRVGRLKL	0	6	6
	WADNDSYNNANQD	1	6	7
	FVVGENSGKPSVRLQL	10	0	10
	YELFHPDEF	1	7	8
	DEIPQPLKPNM	0	4	4
	MLADQEGSDKVAA	0	10	10

Interactions between six PirB^{vp} regions (322-YNRVGRLKL-330, 214-WADNDSYNNANQD-226, 386-FVVGENSGKPSVRLQL-401, 426-YELFHPDEF-434, 290-DEIPQPLKPNM-300 and 409-MLADQEGSDKVAA-421) that were previously reported to bind to PirA^{vp} to form a toxic complex were also analyzed for comparison.

Table S2: Number of residues in the six PirA^{vp}-binding regions of PirB^{vp} protein that are involved in interactions with six chosen oilseed peptides.

Source	Peptide	**PirA^{vp}-Binding Region of PirB^{vp} Protein**						Total Number of Residues
		**214-WADNDSYNNANQD-226**	**290-DEIPQPLKPNM-300**	**322-YNRVGRLKL-330**	**386-FVVGENSGKPSVRLQL-401**	**409-MLADQEGSDKVAA-421**	**426-YELFHPDEF-434**	
Rape	ISYVVQGMGISGR	0	0	0	1	0	2	3
	LTFVVHGHALMGK	0	0	0	0	0	3	3
	QSLGVPPQLGNACNLDNLDVLQPTETIK	0	0	0	3	0	1	4
Sesame	ISTINSQTLPILSQLR	0	0	0	2	0	0	2
Hemp	PQFLVGASSILR	0	0	0	0	0	2	2
	VQVVNHMGQK	0	0	0	0	0	2	2
PirA^{vp}	TIQYQWGAPFMAGGWKVAKSHVVQRDET	0	0	0	3	0	0	3
	WTVEPNGGVTEVDSKHTPIIPEVGRS	0	0	0	4	0	1	5

Interactions between two PirA^{vp} regions (52-TIQYQWGAPFMAGGWKVAKSHVVQRDET-79 and 15-WTVEPNGGVTEVDSKHTPIIPEVGRS-40) that were previously reported to bind to PirB^{vp} to form a toxic complex were also analyzed for comparison.
